# Invasive Goldenrod (*Solidago gigantea* Aiton) as a Source of Natural Bioactive Antimicrobial, Insecticidal, and Allelopathic Compounds

**DOI:** 10.3390/molecules31010126

**Published:** 2025-12-29

**Authors:** Elżbieta Gębarowska, Jacek Łyczko, Anna Kmieć, Paulina Bączek, Kamila Twardowska, Bogdan Stępień

**Affiliations:** 1 Laboratory of Biogeochemistry and Environmental Microbiology, Department of Plant Protection, Wroclaw University of Environmental and Life Sciences, Grunwaldzka 53, 50-357 Wroclaw, Poland; anna.kmiec@upwr.edu.pl; 2 Department of Food Chemistry and Biocatalysis, Wroclaw University of Environmental and Life Sciences, Norwida 25, 50-375 Wroclaw, Poland; jacek.lyczko@upwr.edu.pl; 3 Department of Green Technology, University of Southern Denmark, Campusvej 55, 5230 Odense, Denmark; 4 Division of Entomology, Department of Plant Protection, Wroclaw University of Environmental and Life Sciences, Plac Grunwaldzki 24a, 50-363 Wroclaw, Poland; paulina.baczek@upwr.edu.pl (P.B.); kamila.twardowska@upwr.edu.pl (K.T.); 5 Institute of Agricultural Engineering, Wroclaw University of Environmental and Life Sciences, Chełmońskiego Street 37a, 51-630 Wroclaw, Poland; bogdan.stepien@upwr.edu.pl

**Keywords:** *Solidago gigantea*, invasive plant, phytochemistry, bioactive compounds, antimicrobial activity, insecticidal activity, allelopathy, sustainable agriculture

## Abstract

Goldenrod (*Solidago gigantea* Aiton) is a highly invasive species in Europe (e.g., Poland, Germany, and the Czech Republic) whose secondary metabolites can serve as potential sources of bioactive compounds. This study evaluated the phytochemical profile of *S. gigantea* extracts and evaluated their antibacterial, insecticidal, and phytotoxic activities. The extracts were found to be rich in flavonoids (TFC = 101 mg QE/g) and phenolics (TPC = 175 mg GAE/g), with chlorogenic acid and rutin as dominant constituents. Strong antibacterial activity was observed against Gram-positive bacteria, particularly *Staphylococcus* spp. (MIC_90_ = 2.3 mg/mL; MBC = 5 mg/mL), while Gram-negative bacteria were less sensitive, with moderate susceptibility in *Rhizobium radiobacter* and *Pseudomonas syringae*. The extract exhibited fungistatic activity against all tested filamentous fungi, with *Fusarium* species being the most sensitive (49–56% growth inhibition at 10 mg/mL). Insecticidal assays demonstrated significant mortality of *Tribolium confusum* adults at 2.5–7.0 mg/mL and feeding inhibition at concentrations as low as 0.5 mg/mL. Seedling growth tests showed dose-dependent effects—from mild suppression to moderate stimulation, varying by plant species. Foliar application revealed both stimulatory and inhibitory effects, with the strongest biomass reduction in cress at 10 mg/mL (−45%). These findings indicate that *S. gigantea* extracts possess potent antibacterial, antifungal, insecticidal, and allelopathic activities. Their concentration-dependent effects on pathogens and plants highlight potential applications in sustainable agriculture, including natural crop protection and integrated pest management.

## 1. Introduction

The search for safe and effective alternatives to synthetic pesticides has become a key priority in modern agriculture due to increasing pathogen resistance and the environmental and health risks associated with conventional agrochemicals [1,2,3]. In the European Union, the phasing out of older pesticides and the implementation of sustainability-oriented regulations, such as Directive 2009/128/EC and the “Farm to Fork” strategy, further accelerate the demand for natural plant-derived products with protective potential. At the same time, antimicrobial resistance (AMR) represents a growing global challenge across human and veterinary medicine and crop production, where the extensive use of antibiotics and plant protection agents has led to the emergence of multidrug-resistant pathogens. These pressures highlight the need for innovative, environmentally friendly solutions, including bioactive extracts from plants, which are increasingly recognized as promising tools for integrated pest and disease management [4,5,6,7]. Within this context, bioactive plant extracts represent a sustainable and environmentally friendly solution for crop protection.

Goldenrod (*Solidago gigantea* Aiton) is a perennial herb that belongs to the Asteraceae family, native to North America. It was introduced to Europe in the 17th century as an ornamental plant, but it rapidly became an invasive species in many countries, including Poland, Germany, the Czech Republic, and Scandinavia. Beyond Europe, *S. gigantea* has also become naturalized in Japan, Korea, the Russian Far East, New Zealand, and the Azores [8,9,10,11]. Its presence negatively affects natural plant communities by reducing diversity and abundance while also producing allelopathic compounds that inhibit seed germination and root elongation of neighboring plants. In agricultural systems, these effects can significantly reduce crop yields and increase management costs [12,13,14,15].

Due to these properties, *S. gigantea* represents both an ecological problem and a potential source of valuable bioactive compounds. The bioactive potential of *S. gigantea* is associated with its diverse secondary metabolites. These include flavonoids such as quercetin, kaempferol, and rutin; terpenoids including limonene, α-pinene, and β-caryophyllene; saponins; and phenolic acids such as chlorogenic, caffeic, and rosmarinic acids. These compounds possess antioxidant, anti-inflammatory, antimicrobial, antifungal, antiviral, and insecticidal properties, making *S. gigantea* a promising candidate for the development of natural plant protection products [16,17,18].

In addition, *S. gigantea* and related species have shown insecticidal potential. Benelli [19] reported the effectiveness of essential oils from *S. gigantea* and *S. canadensis* against various insect pests, with *S. canadensis* leaf oil being particularly active. Herrera-Mayorga et al. [20] demonstrated that organic extracts from *Solidago graminifolia* caused mortality of maize pest *Spodoptera frugiperda*. However, the insecticidal activity of methanol or aqueous extracts of *S. gigantea* has not been evaluated, motivating experiments on storage pest *Tribolium confusum*.

The use of goldenrod-derived extracts could offer environmentally friendly alternatives to conventional chemical pesticides, supporting sustainable agriculture initiatives and aligning with European Union policies aimed at reducing synthetic pesticide use [21,22].

Our study provides a comprehensive, multidimensional evaluation of *S. gigantea* extracts, simultaneously assessing their antimicrobial activity against plant pathogens, insecticidal effects on agricultural pests, and allelopathic impacts on economically important crop species. The study aims to evaluate the potential of *S. gigantea* as a source of naturally occurring bioactive compounds for applications in agriculture, plant protection, and pest management while also considering its phytotoxic and growth-stimulating effects. Chemical profiling of the extract and assessments of antibacterial, antifungal, insecticidal, and phytotoxic activities were conducted to support this integrated approach.

## 2. Results

### 2.1. Chemical Profiling

As the main phytochemical groups present in *S. gigantea*, flavonoids and phenolic acids were investigated. The total flavonoid content (TFC) and total phenolic content (TPC) were determined by the aluminum chloride method and the Folin–Ciocalteu method, respectively. The TFP was determined as 101.15 ± 0.83 mg QE/g, while the TPC was 175.47 ± 1.15 mg GAE/g.

In terms of compound identification, the whole list of flavonoids and phenolic acids is given in Table 1. The identification was limited to the compounds available in the standard mixture of phenolic acids, alcohols, and polyphenols: standard flavonoid mixtures MetaSci (Toronto, ON, Canada). Chlorogenic acid (58%) and rutin (55%) were the dominant compounds in the phenolic acid and flavonoid groups, respectively.

### 2.2. Microbial Assay

**Figure 1:**
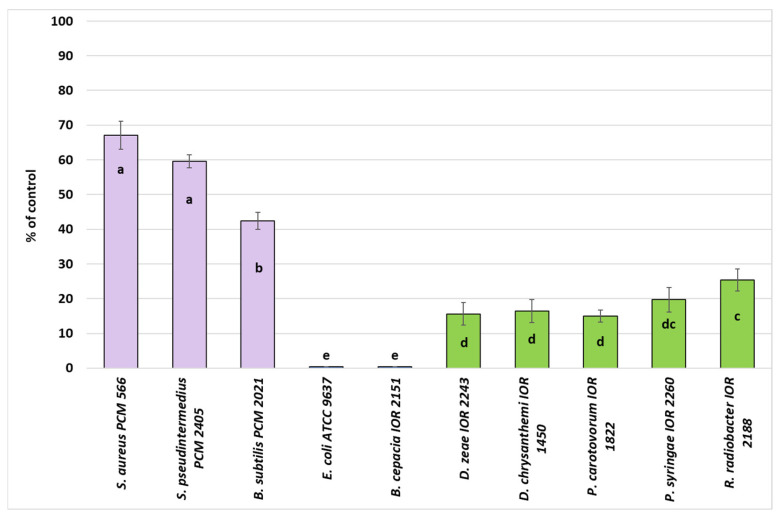
Inhibition coefficient (H) of bacterial cell growth relative to the control (100%). Bars marked with the same letter do not differ significantly (Tukey HSD, *p* < 0.05).

Preliminary evaluation of the antibacterial activity of the *S. gigantea* extract was performed using the agar disc diffusion method, and the results were expressed as the inhibition coefficient (H) of bacterial growth relative to the control (Figure 1). The extract exhibited variable inhibitory activity against the tested bacterial strains. Gram-positive bacteria were more susceptible (H = 42–67%) than Gram-negative bacteria (H = 0–25%). Among the Gram-positive strains, *Staphylococcus aureus* PCM 566 and *Staphylococcus pseudintermedius* PCM 2405 showed the highest growth inhibition, with H values of 67% and 60%, respectively. In contrast, among the plant pathogens, *Rhizobium radiobacter* IOR 2188 (H = 25%) and *Pseudomonas syringae* IOR 2260 (H = 20%) were the most sensitive, while *Dickeya zeae* IOR 2243, *D. chrysanthemi* IOR 1450, and *Pectobacterium carotovorum* IOR 1822 exhibited low inhibition (H = 15–16%).

No inhibitory effect was observed against *Escherichia coli* ATCC 9637 or *Burkholderia cepacia* IOR 2151 at the tested concentration (500 µg/disc).

The results with respect to the antibacterial effect of *S. gigantea* extract against the tested plant and mammalian pathogens are presented in Table 2. The extract exhibited varying bacteriostatic (MIC_50_ and MIC_90_) and bactericidal (MBC) activity against the tested pathogens.

**Figure 2:**
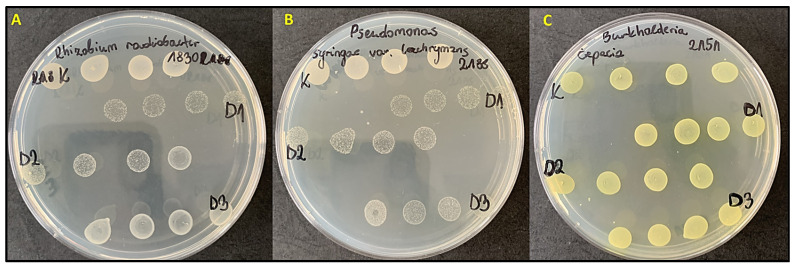
Example of reduced inoculum density (number of bacterial cells) after prior exposure to the goldenrod extract (**A**,**B**) or no effect (**C**). (**A**) *Rhizobium radiobacter* IOR 2188 and (**B**) *Pseudomonas syringae* pv. *lachrymans* IOR 2183 show reduced colony size and density following prior exposure to the extract, indicating a bacteriostatic rather than bactericidal effect. (**C**) *Burkholderia cepacia* IOR 2151 exhibits no visible changes in colony growth, suggesting resistance of this strain to the tested extract.

In the group of plant pathogens, the lowest MIC_50_ and MIC_90_ values were observed for *Rhizobium radiobacter* IOR 2188 (1.0 and 3.9 mg/mL, respectively) and *Pseudomonas syringae* IOR 2260 (1.2 and 4.6 mg/mL, respectively), indicating the high sensitivity of these strains. Moderate sensitivity was found in *Pseudomonas syringae* var. *lachrymans* and *Dickeya zeae*, for which 50% growth inhibition occurred in the concentration range of 4.5–5.2 mg/mL. The lowest susceptibility to the extract was observed for *Burkholderia cepacia* IOR 2151, with MIC_50_ and MIC_90_ values of 10.3 mg/mL and 16.6 mg/mL, respectively. No bactericidal activity (MBC) was observed against plant pathogens within the tested concentration range (0.625–10 mg/mL). However, reductions in cell growth were detected at 5 and 10 mg/mL (Figure 2).

Among mammalian pathogens, the lowest MIC_50_ value was obtained for endospore-forming rod *Bacillus subtilis* PCM 2021 (1.2 mg/mL), followed by *Staphylococcus aureus* PCM 566 (2.2 mg/mL) and *Staphylococcus pseudintermedius* PCM 2405 (3.8 mg/mL). The extract demonstrated bactericidal activity against Gram-positive bacteria at a dose of 10 mg/mL. In contrast, no inhibitory or bactericidal effect was observed for Gram-negative rods, including *Escherichia coli* PCM 2561, for which both MIC and MBC exceeded 10 mg/mL.

These findings indicate that the extract has selective antibacterial properties that are particularly effective against Gram-positive pathogens.

The growth inhibition/stimulation coefficient (H/S%) of filamentous fungi and oomycote exposed to *S. gigantea* extract is presented in Table 3. The extract was tested at doses of 2.5, 5, and 10 mg/mL using the poisoned medium technique on Czapek-Dox medium.

The fungistatic effect of the *S. gigantea* extract varied and ranged from 2 to 56%, depending on the extract concentration (X) and the fungal or oomycote species (Y). A significant effect of both extract dose (X) and fungal species (Y) was observed (Tukey HSD, *p* = 0.05). The fungistatic activity increased consistently with extract concentration (X). Mean inhibition increased with dose (X), from 34.2% at 2.5 mg/mL to 43.8% at 10 mg/mL, with all means significantly different from each other (X: C < B < A). The highest inhibition was achieved at 10 mg/mL, reaching up to 56% in *Fusarium culmorum* (strain Fc16). Saprotrophic fungi of the genus *Mucor* also showed a clear dose-dependent response (29%, 44%, and 55% at D1–D3, respectively). In contrast, *Trichoderma harzianum* exhibited stable inhibition across all doses (44–47%), with no significant differences between treatments.

Highly significant differences in the effect of fungistaticity between tested species were recorded (Y: F < E < D < C < B < A). The strongest fungistatic effect was observed against *F. culmorum*, especially in strains Fc5, Fc6, and Fc16, in which 49–56% growth inhibition was observed across doses. For *F. graminearum*, the inhibition exceeded 50% only at the highest dose (D3). The weakest inhibition was recorded for *B. cinerea* (Y = 3%) and *Rh. solani* F93 (Y = 13%). At the highest dose, growth inhibition was only 8% and 23%, respectively, indicating low susceptibility. The radial growth of the oomycote of *Phytophthora cinnamomi* IOR 2080 was dose-dependent, with growth reductions ranging from 35.1 ± 0.3% to 40.5 ± 0.3% compared to the control.

No stimulatory effect (S < 0) was observed for any of the tested species. Even *B. cinerea*, which showed an effect in the range of −0.8% to 7.8%, did not exhibit true stimulation.

**Figure 3:**
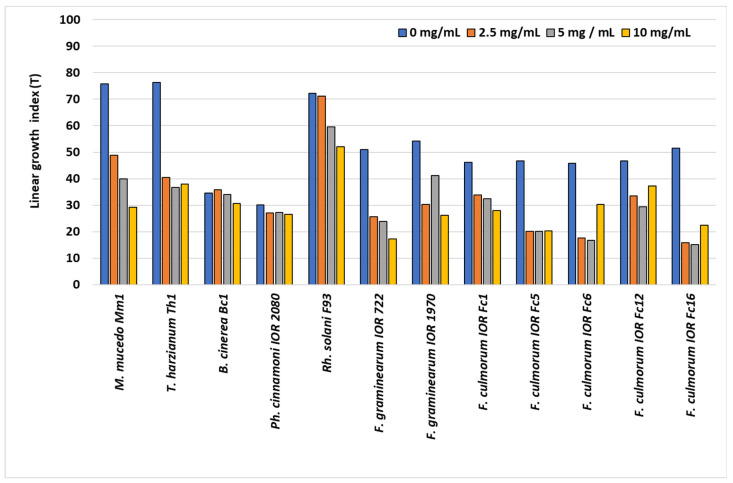
Linear growth rate index (T) of colonies of saprotrophic and polyphagous plant-pathogenic species on Czapek medium supplemented with *Solidago nigrum* extract.

The linear growth rate index (T) of the saprotrophic and polyphagous plant-pathogenic fungi and oomycote treated with *S. gigantea* extract is illustrated in Figure 3. Growth rates varied significantly according to the tested species and the concentration of the extract.

Saprotrophic fungi of the genus *Mucor* exhibited a clear dose-dependent reduction in growth rate. *M. mucedo* Mm1 showed a decrease from T = 76 in the control to T = 29 at the highest extract concentration (10 mg/mL). In *T. harzianum* Th1, the growth rate decreased from T = 76 to 38 but remained relatively stable under all extract doses, indicating dose-independent moderate inhibition.

Among polyphagous plant pathogens, *Ph. cinnamoni* IOR 2080 and *B. cinerea* Bc1 showed minimal changes in growth rate compared to controls (T range: 27–36), indicating low sensitivity to the extract. *Rh. solani* F93 exhibited a moderate decrease in T from 72 to 52 at the highest dose.

*Fusarium* species showed variable responses. *F. graminearum* IOR 722 and 1970 showed a strong reduction in growth rate (T = 51 → 17 and T = 54 → 26, respectively). Among *F. culmorum* strains, the most pronounced inhibition was observed in Fc5 and Fc16, with T decreasing to 15–20, representing approximately a two- to three-fold reduction compared to controls. Other strains of *F. culmorum* (Fc1, Fc6, and Fc12) exhibited intermediate responses, with partial recovery in some cases at 10 mg/mL.

No stimulatory effect of the extract on the colony growth rate was observed for any tested species. In general, the extract exerted the strongest inhibitory effect on *Mucor* and selected *Fusarium* strains, moderate inhibition on *Rhizoctonia*, and minimal impact on *Phytophthora* and *Botrytis*.

**Figure 4:**
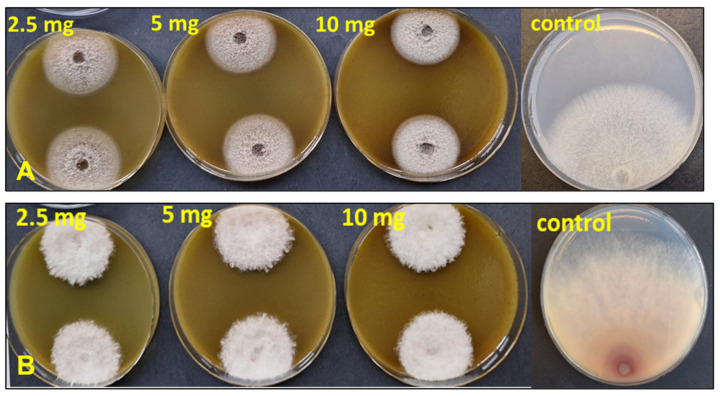
Radial growth rate of *Ph. cinnamomi* 2080 (**A**) and *F. culmorum* Fc12 (**B**).

The effect of the extract in inhibiting the growth rate of the tested isolates is shown in Figure 4.

**Figure 5:**
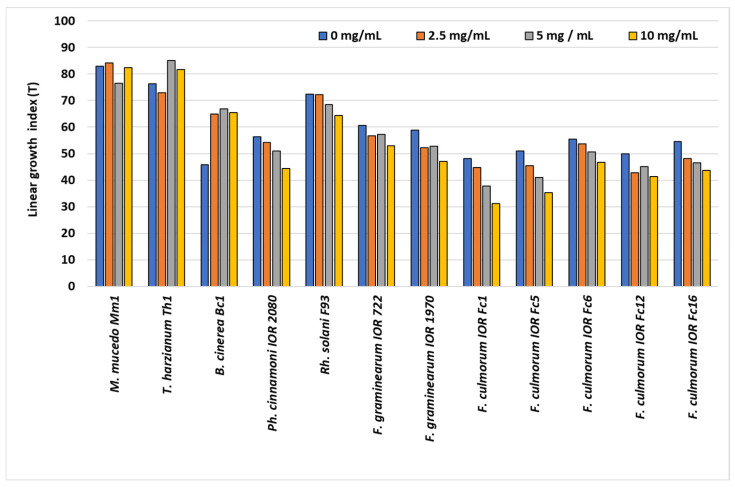
Growth inhibition coefficient (H) of saprotrophic and polyphagous plant-pathogenic fungi on Czapek-Dox medium without *Solidago gigantea* extract after reisolation.

To assess the biocidal activity of the extract (Table 4), the tested species of fungi and oomycote were reisolated on Czapek-Dox control medium (without *S. gigantea* extract). The study confirmed that goldenrod extract has no fungicidal effect, as the tested strains were able to grow again. However, a subsequent fungistatic or biostimulating effect was observed (Table 4 and Figure 5), depending on the concentration of the extract (X) and the fungal species (Y). Goldenrod extract showed a stimulating effect on *B. cinerea* Bc1 (Y ≈ 32%). *T. harzianum* Th1 growth was also stimulated, but only at the 5 mg/mL dose (12%). In the case of fungi of the *Fusarium* genus and oomycete *Ph. cinnamoni*, mycelial growth inhibition (Y) was observed at a level of 18–29% at a dose of 10 mg/mL. The strongest inhibition was observed for isolates *F. culmorum* Fc1 and Fc5 (H = 29 and 24%, respectively).

Analysis of the growth coefficient (T) showed different responses of individual species depending on the extract dose (Figure 5). The growth rate was recorded in the range of T = 31–85. Saprotrophs *M. mucedo* Mm1 and *T. harzianum* Th1 maintained a high growth rate, regardless of dose (T = 77–84 and T = 73–85, respectively), indicating no permanent growth inhibition.

Among polyphagous plant pathogens, responses to the extract varied. *B. cinerea* Bc1 exhibited clear growth stimulation compared to the control (T = 46 → 65–67). *Ph. cinnamoni* IOR 2080 and *Rh. solani* F93 showed moderate decreases in growth rate at the highest dose (T = 44 and 64, respectively), indicating reversible inhibition.

Within the *Fusarium* species, the strongest reduction in growth rate was observed for *F. culmorum* Fc1 and Fc5 (T = 48 → 31; 51 → 35), while the remaining strains (Fc6, Fc12, and Fc16) showed a moderate decrease (T = 55 → 44). *F. graminearum* IOR 722 and IOR 1970 also showed reductions in growth rate in the ranges of T = 61 → 53 and 59 → 47.

In conclusion, goldenrod extract did not show biocidal activity against any of the tested fungi or oomycote strains. The application of the extract caused a reversible, dose- and species-dependent reduction in the growth rate of the mycelium, and the only case of a stimulatory effect was an increase in the growth rate of *B. cinerea* Bc1.

### 2.3. Insecticidal Activity

The analyses indicate how different extract concentrations affect the mortality and feeding (loss of wheat-wafer mass) of female and male confused flour beetles (*Tribolium confusum*). Statistical analysis showed different effects depending on the concentration of the extract and the sex of the pest used for biotests.

**Figure 6:**
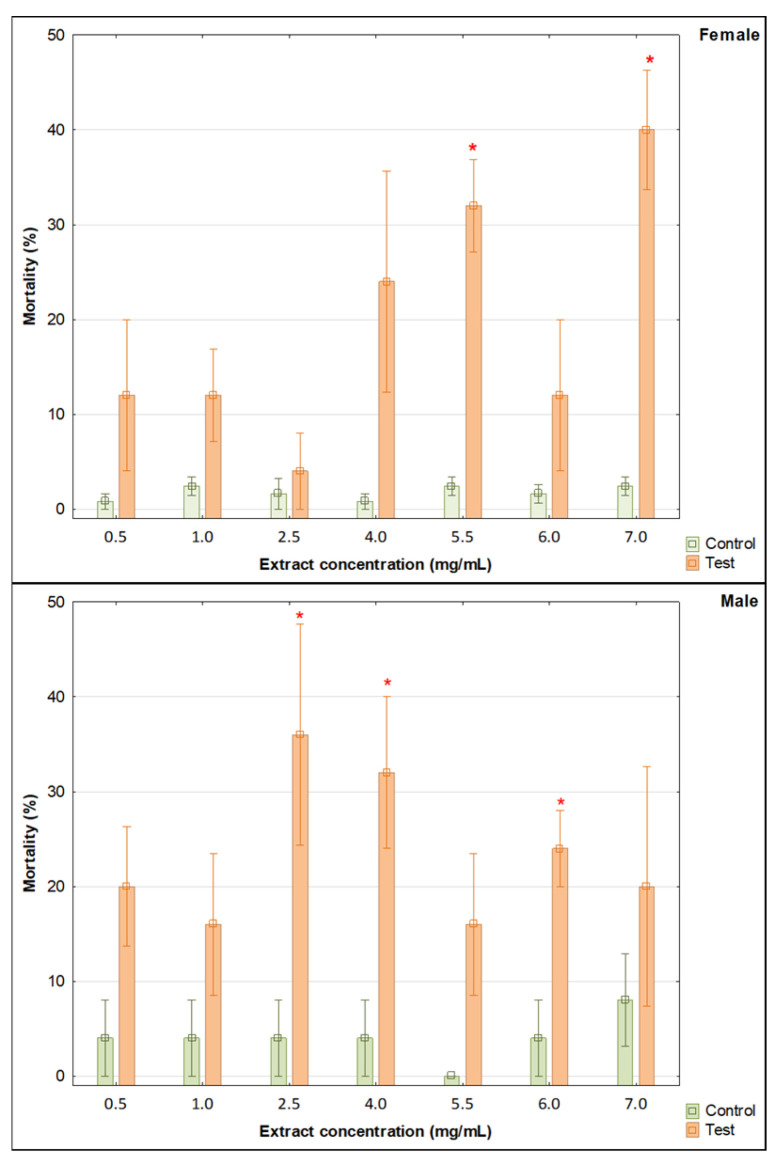
Mortality of *T. confusum* females and males under the influence of methanol extracts of various concentrations (Mean ± SE). Significant differences between pairs (control vs. tested concentration of the *Solidago gigantea* extract) were estimated using the Mann–Whitney U test, marked with * (*n* = 5).

Specifically, statistical analysis showed that female mortality was significantly higher at doses of 5.5 and 7.0 mg/mL compared to the control (Table 5, Figure 5). For all other doses, female mortality was also higher compared to the control, but these differences were not significant. There was no significant effect of the tested extract on the feeding behavior of the females (loss of wheat-wafer mass) (Table 5, Figure 6).

**Figure 7:**
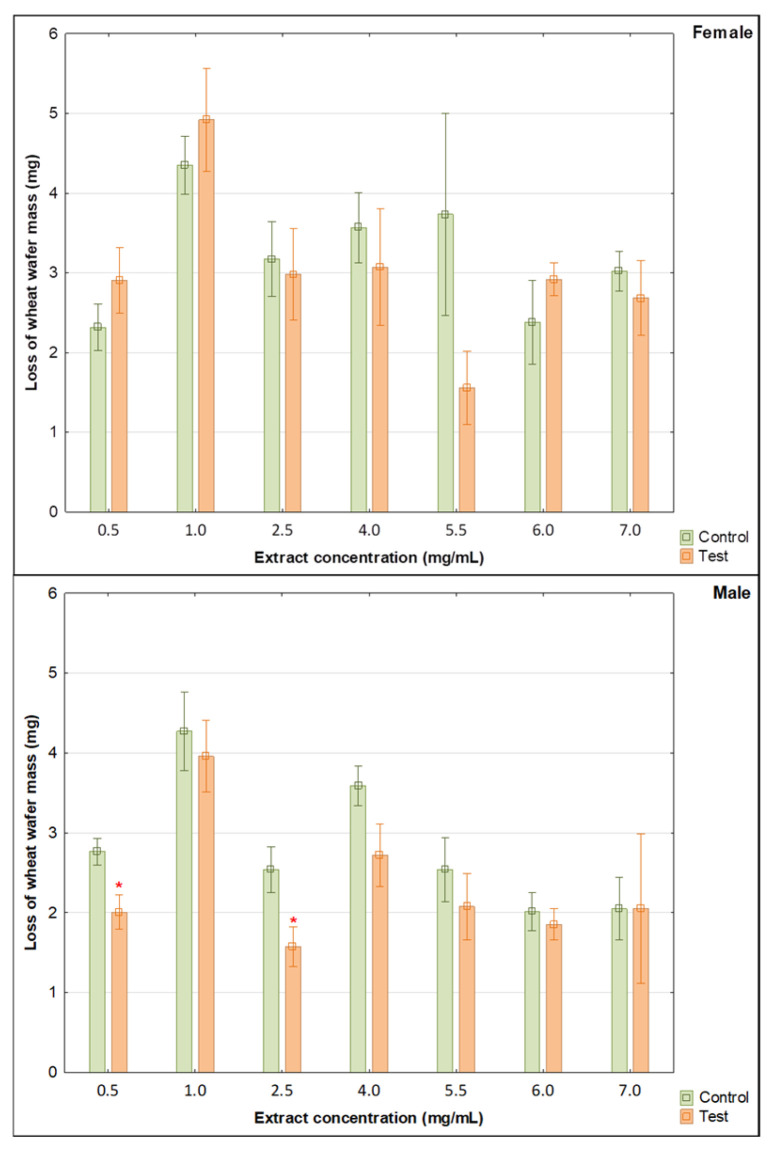
Loss of wheat-wafer mass as a result of feeding by *T. confusum* females and males under the influence of methanol extracts of various concentrations (Mean ± SE). Significant differences between pairs (control vs. tested concentration of the *Solidago gigantea* extract) were estimated using the Mann–Whitney U test, marked with * (*n* = 5).

For males, mortality was significantly higher for doses of 2.5, 4.0, and 6.0 mg/mL compared to the control (Table 5 and Figure 7). However, the mortality was not significantly higher than in the control for all other extract concentrations. Furthermore, significant feeding inhibition (wheat-wafer mass loss) was observed in males (Table 5 and Figure 7). In more detail, the loss of mass of the wheat wafer was significantly lower compared to the control using 0.5 and 2.5 mg/mL doses of the extract. When doses of 1.0, 4.0, 5.5, and 6.0 mg/mL were used, the loss of wheat-wafer mass was also greater in the control (no significant differences).

### 2.4. Effects of Solidago Gigantea Aqueous Extract on Seed Germination and Plant Growth

**Figure 8:**
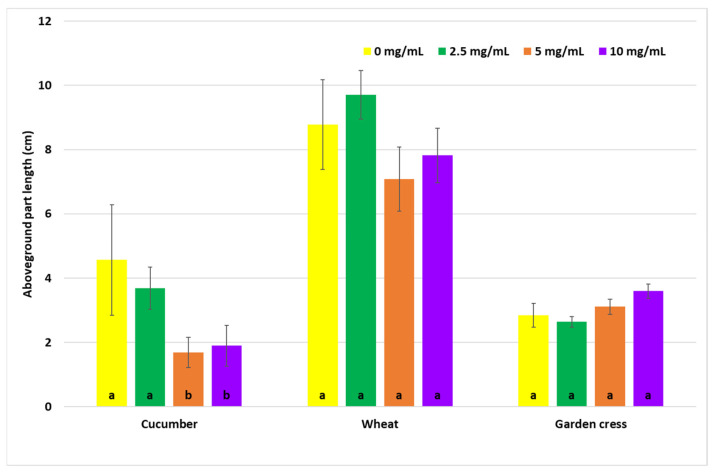
Effect of seed treatment with aqueous goldenrod extract on plant growth (aboveground part length, cm). The values of bars marked with the same letters are not significantly different according to Tukey’s HSD test (*p* = 0.05).

The effect of the aqueous *S. gigantea* extract on the growth of 7-day-old seedlings of the tested plant species is shown in Figure 8. In general, the doses of the applied extract did not significantly affect the growth of the seedling. A twofold reduction in cucumber seedling growth compared to the control was observed at doses of 5 and 10 mg/mL. In contrast, the growth of cress seedlings remained at a similar level (2.8–3.6 cm), although the highest dose exhibited a stimulatory effect, increasing seedling length by 27%.

**Figure 9:**
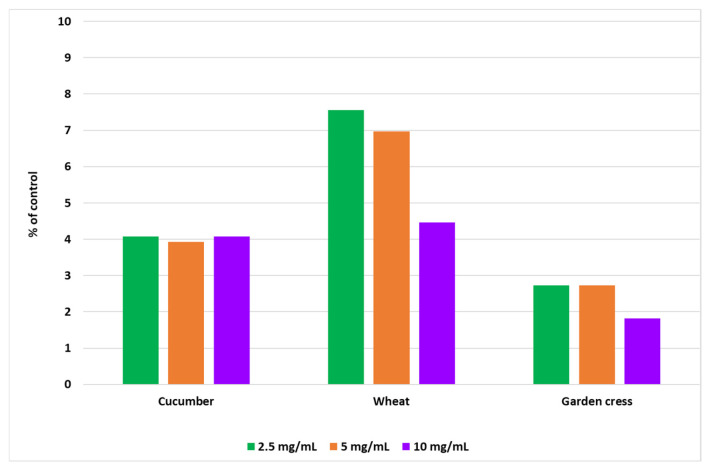
Effect of aqueous goldenrod extract seed treatment on plant dry matter (% of control).

The effect of the aqueous extract of *Solidago* on the dry weight of 7-day-old seedlings of the tested plants is presented in Figure 9. The results are expressed as percentages relative to the control. Application of the *Solidago* extract generally led to a slight reduction in seedling biomass, with the decrease not exceeding 10%. The greatest reduction in dry weight—8%—was observed in winter wheat following the application of the 2.5 mg/mL dose. No stimulatory effect of the extract on biomass accumulation was detected in any of the tested plant species.

The effect of *S. gigantea* extract was assessed using a phytotoxicity index (Table 6 and Table 7) after each spray was applied on the 10th, 15th, and 20th day of plant growth using doses of 2.5, 5, and 10 mg/mL. Both stimulatory and inhibitory effects were observed.

**Figure 10:**
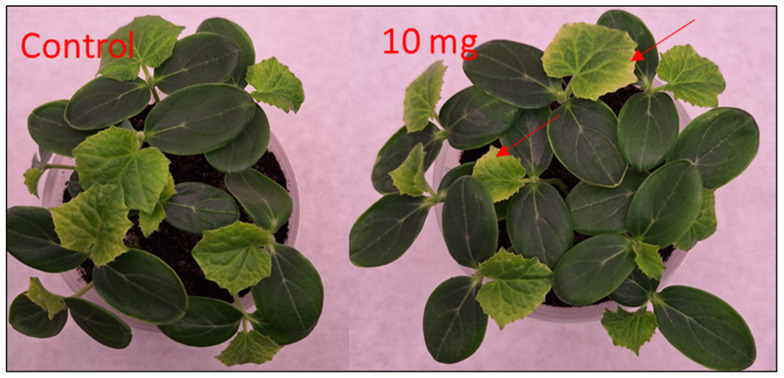
Selected seedlings of field cucumber (*Cucumis sativus*) after foliar treatment with *S. gigantea* extract at different concentrations. Visible yellowing (chlorosis) is highlighted with arrows at the highest dose (10 mg/mL). The figure provides visual confirmation of dose-dependent phytotoxic and growth-modulating effects.

The spray applied on the 10th day of growth caused yellowing of the leaf margins, especially in cucumber plants, particularly at doses of 10 and 5 mg/mL. Subsequent treatments did not induce further phytotoxic effects, and the condition of the plant improved. The discoloration, particularly on the cucumber leaves, disappeared (Figure 10).

After the phytotron experiment was completed, the dry mass of the plants was determined. The dry mass values varied and depended on both the applied extract dose (X) and the plant species (Y) (Table 7), which exhibited stimulating and inhibitory effects on plant growth.

The strongest stimulatory effect relative to the control was observed in cucumber (X = 15%). The phytotoxicity of the extract increased with dose (Y = 108% > 99% > 81). Foliar application at 2.5 mg/mL significantly increased the dry mass of cucumber (by 37% relative to the control), while the 5 mg/mL dose stimulated the growth of winter wheat (by 11%). The greatest biomass reduction was recorded in garden cress at the highest dose of 10 mg/mL (a decrease of 45%), whereas in the other plants, the reduction in dry mass at this dose did not exceed 10%.

Overall, the results indicate that the aqueous extract of *S. gigantea* does not exhibit unequivocal phytotoxicity, and its effects on plants depend on both the plant species and the applied dose. At low concentrations, the extract may demonstrate growth-stimulating properties, whereas higher doses can limit growth, particularly in more sensitive species. These findings suggest the potential use of compounds present in the extract as natural plant growth regulators or biostimulants, although their dose-dependent effects must be carefully considered.

## 3. Discussion

Extracts from invasive plant species such as *S. gigantea* offer a dual benefit: they provide a renewable source of bioactive metabolites while enabling the utilization of ecologically problematic biomass. The results of our LC-MS/MS analysis confirmed that the methanolic extract of *S. gigantea* used in this study is rich in polyphenols, consistent with previous research on *Solidago* species [18,19,20,23]. The total flavonoid content (TFC) and total phenolic content (TPC) fall within the ranges reported for other *Solidago* taxa. For example, Shelepova et al. [23] reported TFC values of 65–175 mg QE/g and TPC values of 204–293 mg GAE/g in *S. canadensis*, while *S. graminifolia* exhibited comparable levels [24]. Fourteen major metabolites were identified, including six phenolic acids, seven flavonoids, and one phenolic alcohol. Chlorogenic acid (CGA) and rutin were the dominant representatives of their respective classes. This composition closely matches that reported for *Solidago* populations from similar climatic regions Shelepova et al. [23], suggesting a shared chemotype in Central European stands.

Allelopathic activity is considered a key contributor to the invasiveness of *Solidago* species, which frequently accumulate high levels of phenolic acids and flavonoids capable of suppressing germination and growth of neighboring plants [25,26,27]. The abundance of CGA, rutin, quercitrin, and quercetin, compounds widely recognized for their antimicrobial and allelopathic activity, provides a mechanistic basis for the biological effects (bacteriostatic, fungistatic, and insecticidal) observed in this study [18,19,20].

Allelopathic assays demonstrated clear species- and dose-dependent responses. Low extract concentrations stimulated growth in cucumber and winter wheat, whereas higher doses inhibited development and caused transient chlorosis in cucumber. Garden cress was strongly inhibited in all treatments. These findings follow the hormesis model typical of allelopathy [28,29], which is driven by phenolic acids and flavonoids known to affect membrane permeability, oxidative stress, nutrient uptake, and hormonal signaling [30].

Integrating the phytochemical profile with biological activity suggests that chlorogenic acid, rutin, quercetin derivatives, and terpenoid lactones present in *S. gigantea* may jointly contribute to its allelopathic effects. These compounds have documented roles as germination inhibitors, growth modulators, microbial growth suppressors, and insect feeding deterrents [31,32], aligning strongly with the multifaceted effects observed in the present study.

Antibacterial assays revealed marked selectivity between Gram-positive and Gram-negative strains. *B subtilis*, *S. aureus*, and *S. pseudintermedius* were found to be highly susceptible (MIC_90_ = 7.5–9.3 mg/mL; MBC 10 mg/mL), consistent with previous studies on *S. gigantea* and purified diterpenoids [18,33]. Gram-negative plant pathogens showed only bacteriostatic responses—especially *P. syringae* and *Rh. radiobacter*—while *E. coli* and *B. cepacia* were largely unaffected, reflecting the protective role of their outer membrane [34].

The extract also showed moderate but broad fungistatic activity. Growth of *Fusarium* spp., *B. cinerea*, *Rhizoctonia solani*, and *Ph. cinnamomic* (oomycete) was reduced by 40–56% at 10 mg/mL, with similar effects on saprotrophs. No fungicidal action or hormetic stimulation occurred. These findings align with earlier reports demonstrating moderate inhibition by *Solidago* extracts without fungal growth promotion [35].

The observed biological effects correspond closely with the known properties of the dominant metabolites. Chlorogenic acid disrupts microbial membranes, affects ion homeostasis, modulates metabolic enzymes, inhibits spore germination, and interferes with quorum sensing [36,37,38,39,40,41,42]. Rutin and quercetin exhibit antibacterial, antifungal, and antiviral activity; synergize with other phenolics; and impair biofilm formation [43,44,45,46,47]. The combined action of these compounds explains the extract’s activity spectrum.

The extract showed significant insecticidal effects against *Tribolium confusum*. Male mortality occurred at 2.5–6 mg/mL, while females responded at 5.5–7 mg/mL. Feeding inhibition was significant only in males. Comparable studies demonstrate similar activity for other *Solidago* species, with solvent choice, plant organ, insect species, and life stage strongly influencing toxicity [21,22,48,49,50]. CGA, quercetin, and rutin likely contribute to the observed activity, as these compounds are known to influence insect feeding and survival in several species [22,51,52,53,54,55].

Overall, the allelopathic, antimicrobial, and insecticidal properties of *S. gigantea* extracts indicate the multifunctional bioactivity of this invasive species. These effects likely result from the combined action of phenolic acids and flavonoids and are consistent with known mechanisms such as membrane disruption, modulation of oxidative stress, and interference with microbial communication systems. The extract’s activity profile, moderate antifungal effects, noticeable activity against Gram-positive bacteria, limited effects on Gram-negative strains, and measurable insecticidal activity, suggests that *S. gigantea* biomass could serve as a potential resource for sustainable agriculture, integrated pest management, and ecological valorization. Further research should aim to identify synergistic metabolites, optimize extraction methods, and expand testing against pathogen and pest to better assess its practical applications and environmental relevance.

## 4. Materials and Methods

### 4.1. Plant Material and Extract Preparation

Plant material of *Solidago gigantea* A. was collected in September 2023 at the flowering stage in the municipality of Wisznia Mała, Poland (51.213353, 17.046135). Plants were transported directly after harvest from the field to the laboratory of the Institute of Agricultural Engineering at the Wrocław University of Environmental and Life Sciences. Before drying, the material was stored under refrigerated conditions (4 ± 1 °C) and protected from light to minimize degradation processes. Before the drying process, the raw material was cut into 20 ± 2 mm long sections. The sublimation drying process was carried out using a Free-Zone 4.5 L Plant installation (Labconco, Fort Scott, KS, USA). The plant material was pre-frozen outside the drying chamber at a temperature of −20 °C, with a freezing rate of 1 °C/min, in order to avoid the undesirable effect of self-freezing. Immediately after the freezing process, the samples were placed on trays in the drying chamber, where the pressure was reduced to 100 Pa. According to literature reports, the contact method of supplying heat to the sample at a high heating-plate temperature shortens the drying time but may adversely affect the quality of the dried product. Therefore, a compromise solution was adopted, using the contact method of heat transfer and a low heating-plate temperature of 22 °C.

Dried plants were ground in a grinder (IKA POL M20 Universal Mill, Staufen, Germany) and extracted with absolute methanol (ratio: 1:5, *w*/*v*). Further on, the mixture was shaken for 60 min on a rotary shaker (125 rpm). Finally, the crude extracts were filtered and dried with a rotary evaporator (VWR IKA, Ulm, Germany). The dry extracts were weighed and stored at 4 °C until further testing [56].

### 4.2. Chemical Analysis

#### 4.2.1. LC-MS/MS Analysis

For flavonoid and phenolic acid identification, the plant methanolic extract was prepared. Briefly, 50 g of powdered, aerial plant parts were suspended in 500 mL of 80% [*v*/*v*] methanol (Sigma Aldrich, Steinheim, Germany) for 24 h under constant shaking (120 rpm) based on previously applied protocols [56]. After the extraction time, the solvent was separated from the extracted material, and the procedure was repeated three times in total. After collecting 3 portions of extract, the solvent was evaporated with a vacuum rotary evaporator, and for further analyses, samples with an extract concentration of 50 mg/mL were prepared by dissolving the extract with an accuracy of ±0.005 g in Chromasolv-grade methanol (Sigma Aldrich, Steinheim, Germany), diluting it 100 times and filtering through Phenex-RC syringe filters (Phenomenex, Torrance, CA, USA). Before the analysis, 100 µg of luteolin (Sigma-Aldrich, Steinheim, Germany) was added to each sample as an internal standard (the presence of luteolin in the sample was determined with an earlier test). The analyses were run in triplicate on an LCMS-8045 instrument with an ESI-type ion source (Shimadzu, Kyoto, Japan) equipped with a Kinetex 2.6u C18 100A 100 × 3.0 mm column with a 3 mm ULTRA shell (Phenomenex, Torrance, CA, USA).

The mobile phase used for chromatographic separation consisted of 0.1% aqueous formic acid (A) and methanol (B). A solvent gradient was applied—from 10% to 20% B in 0–5 min, 60% B in 5–10 min, and 60 to 10% B in 10–13 min—and kept for 4 min to stabilize the analytical conditions. The flow rate and injection volumes were 0.35 mL/min and 10 µL, respectively. The column temperature was set to 35 °C.

The identification of flavonoids and phenolic acids was based on the MRM mode (details in Table 1). Operational conditions: nitrogen for nebulization (flow 3 L/min) and desolvation (temperature 526 °C); heating and drying gas flow of 10 L/min; and interface, DL, and heating-block temperatures of 300, 250, and 400 °C, respectively.

#### 4.2.2. Total Flavonoid Content (TFC) and Total Phenolic Content (TPC)

The TFC and TPC were determined with the colorimetric spectroscopic method. Briefly, for TFC, 1 mL of extract with a concentration of 100 ug/mL in methanol (Sigma Aldrich, Steinheim, Germany) was mixed with 0.2 mL aluminum chloride solution (10% *w*/*v*) and potassium acetate (1 M) and 5.6 mL distilled water and left for 30 min. Thereafter, the samples were analyzed with a Cintra 303 spectrometer (GBC Scientific Equipment Ltd., Dandenong, Australia) at 415 nm. The TFC was expressed as quercetin equivalents.

The TPC was determined with the Folin–Ciocalteu method. A volume of 1 mL of plant extract (100 ug/mL in methanol) was mixed with 2.5 mL of 10% [*w*/*v*] Folin–Ciocalteu reagent and kept for 5 min. Thereafter, 2 mL of sodium carbonate (75% *w*/*v*) was added, and the sample was incubated in a water bath for 10 min at 50 °C. After that, the samples were subjected to absorbance read with a Cintra 303 spectrometer (GBC Scientific Equipment Ltd., Dandenong, Australia) at 765 nm. The results were expressed as mg of gallic acid equivalents (GAE).

### 4.3. Antimicrobial Assay

#### 4.3.1. Test Microorganisms

The obtained extracts of *S. gigantea* were tested against a wide range of microorganisms, including Gram-positive cocci (*Staphylococcus aureus* PCM 566 and *Staphylococcus pseudintermedius* PCM 2405), Gram-positive endospore-forming rods (*Bacillus subtilis* PCM 2019), Gram-negative rods (*Escherichia coli* ATCC 9637 and plant-pathogenic bacteria such as *Burkholderia cepacia* IOR 2151, *Dickeya zeae* IOR 2243, *Dickeya chrysanthemi* IOR 1452 (pv. *Erwinia chrysanthemi*), *Pectobacterium carotovorum* IOR 1822, (pv. *Erwinia carotovora*), *Pantoea agglomerans* IOR 2187, *Pseudomonas syringae* IOR 2260, *Pseudomonas syringae* var. *lachrymans* IOR 2183, and *Rhizobium radiobacter* IOR 2188); and polyphagous plant-pathogenic fungi (*Fusarium culmorum* IOR strains Fc5 (79), F6 (1596), and Fc12 (8); *F. culmorum* Fc1 and Fc16; *Fusarium graminearum* IOR strains 722 and 1970; *Botrytis cinerea* Bc1; *Phytophthora cinnamoni* IOR 2080 (oomycete); *Rhizoctonia solani* F93; and saprotrophic fungi *Trichoderma harzianum* Th1 and *Mucor mucedo* Mm1).

These strains came from the Polish Collection of Microorganisms (PCM, Institute of Immunology and Experimental Therapy, Polish Academy of Sciences, Wroclaw, Poland) and the Culture Collection of Plant Pathogens at the Institute of Plant Protection (IOR, Poznań, Poland). Filamentous fungi were obtained from IOR or from our own collection. Cultures of bacteria were maintained on LBA (Luria Broth Agar, Sigma-Aldrich, St. Louis, MO, USA), while fungi were maintained on PDA (Potato Dextrose Agar, Sigma-Aldrich, St. Louis, MO, USA), and both were stored on slants at 4 °C.

#### 4.3.2. Agar Disc Diffusion Assay

The antibacterial activity of *Solidago gigantea* extracts was assessed using the agar disc diffusion method according to Matuschek et al. (2014) [57]. A 100 μL aliquot of bacterial suspension (1.5 × 10^8^ CFU/mL) was spread on Mueller–Hinton agar (MHA) for mammalian pathogens or Luria broth agar (LB) for plant pathogen. The turbidity of the bacterial strains was standardized to 0.5 McFarland (spectrophotometer VIS-723G, Rayleigh, Beijing, China). Paper discs (Whatman no. 1, England, 5 mm diameter) were placed on the agar surface and impregnated with 30 μL of stock solutions of 500 µg/disc. A 10% DMSO solution was used as a solvent and negative control, while filter discs impregnated with 30 µg/mL gentamicin (Sigma-Aldrich, USA) served as the positive control. Plates were incubated at 37 °C for 24 h for mammalian pathogens and at 28 °C for plant pathogens. Antimicrobial activity was evaluated by measuring the inhibition-zone diameters (in mm) with a caliper and subtracting the disk diameter (5 mm) to obtain the actual inhibition-zone size. Each assay was performed in triplicate, and mean values are presented (Figure 1). The results are expressed as the inhibition coefficient (I) of bacterial growth according to Formula (1):

(1) $$I\%\;=\;\frac{C-S}{C}\times 100$$

where *C* is the inhibition zone of the positive control and *S* is the inhibition zone obtained for the *S. gigantea* extract.

#### 4.3.3. Determination of Minimum Inhibitory Concentration (MIC) and Minimum Bactericidal Concentration (MBC)

The minimum inhibitory concentrations (MICs) were determined by a serial dilution method [58] in 96-well tissue culture plates (VWR European, Leuven, Belgium). Bacterial species were cultured on Mueller–Hinton agar (MHA; Sigma-Aldrich, St. Louis, MO, USA) at 36 °C, while phytopathogens were incubated on Luria broth (LB; Sigma-Aldrich, St. Louis, MO, USA) at 28 °C for 20 h. After incubation, bacterial suspensions were prepared in 0.9% NaCl, and their turbidity was standardized to 0.5 McFarland using a VIS-723G spectrophotometer (Rayleigh, Beijing, China). The final bacterial inoculum density was approximately 5 × 10^5^ CFU/mL.

Dry extracts were dissolved in 10% dimethyl sulfoxide (DMSO; Sigma-Aldrich, St. Louis, MO, USA) and prepared in concentrations ranging from 10 to 0.625 mg/mL. A 10% DMSO solution was used as the negative control.

**Figure 11:**
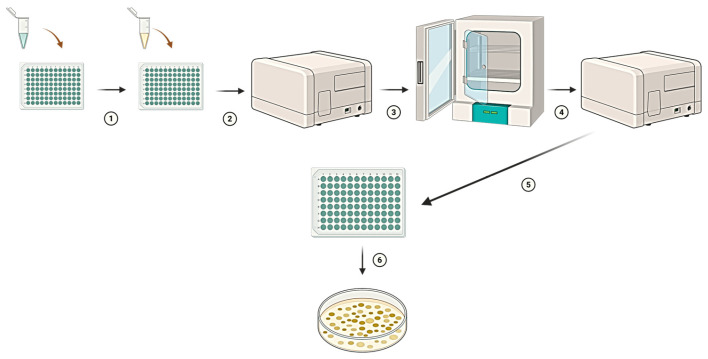
Schematic procedure for MIC and MBC determination. (1) Preparation of serial extract dilutions in a 96-well microtiter plate. (2) Addition of bacterial inoculum to each well containing the extract. (3) Recording of baseline optical density (OD) using a microplate spectrophotometer. (4) Incubation of plates for 20 h at 28/36 °C. (5) Post-incubation OD measurement (MIC determination). (6) Aliquots from wells without visible growth were plated onto agar to determine bactericidal activity (MBC determination).

After adding the bacterial inoculum, plates were incubated at 37 °C for bacteria and 28 °C for phytopathogens for 20 h. Bacterial growth was monitored by measuring the optical density (OD) at 590 nm using a microplate photometer (Multiscan Go, Thermo Scientific, Waltham, MA, USA). Each experiment was performed in three independent series with four technical replicates per treatment (see Figure 11).

The MIC_90_ was defined as the lowest concentration of extract that inhibited ≥90% of bacterial growth relative to the positive control. The percentage of growth inhibition was calculated according to Formula (2):

(2) $$\%Inhibition\;OD=\;\frac{{OD}_{C}-{OD}_{S}}{{OD}_{C}}\times 100$$

where *OD_C_* is the mean optical density of the positive control and *OD_S_* is the optical density of the test sample.

The minimum bactericidal concentration (MBC) was defined as the lowest extract concentration that killed ≥99% of bacterial cells. To determine MBC, cultures from wells showing no visible growth were inoculated onto MHA plates and incubated under the same conditions.

#### 4.3.4. Antifungal Activity

Antifungal activity was evaluated using the Poisoned Food Technique [59] and modified according to our previous study Gębarowska et al. [56]. The extract, dissolved in 10% DMSO, was added to the liquefied Čapek agar medium (cooled to 56°C) to obtain final concentrations of 10.0, 5.0, and 2.5 mg/mL (D1–D3). The medium was poured into Petri dishes and allowed to solidify. After solidification, the medium was inoculated with two 5 mm diameter discs cut from the actively growing margin of a one-week-old fungal colony, which were placed opposite each other on the plate. After 24 h of incubation, the mycelial discs were aseptically removed. The control contained the solvent (10% DMSO) at the highest concentration. All experiments were performed in four repetitions. The radial colony growth was measured daily until the control colonies fully covered the surface of the Petri dish.

Additionally, to determine the fungicidal activity of the goldenrod extract, the tested fungi were re-isolated onto Czapek–Dox control medium (without the extract) after the experiment was completed.

The results are expressed as the linear growth index and the colony growth inhibition or stimulation coefficients. The linear growth index is calculated using Formula (3) [60]:

(3) $$T=\frac{A}{D}+\frac{b_{1}}{d_{1}}+\ldots +\frac{b_{x}}{d_{x}}$$

where

T—linear growth index;

A—average measurements of colony diameter (mm);

D—duration of the experiment (days);

b1–bx—the diameter increase in the colony since the last measurement (mm);

d1–dx—the number of days since the previous measurement.

The coefficient of radial growth inhibition (I) or stimulation (S) of the fungi was calculated using the modified Abbott Formula (4) [60]:

(4) $$I/S\;(\%)\;=\;\frac{C-S}{C}\times 100$$

where

*I*/*S*—inhibition (*I*) or stimulation (*S*) of radial mycelial growth (%);

*C*—colony diameter on the control plate (mm);

*S*—colony diameter on the plate containing the extract.

### 4.4. Insecticidal Assays

The insecticidal activity of *Solidago gigantea* extract was determined in one species of pest storage: confused flour beetle (*Tribolium confusum* Duv.) (Coleoptera, Tenebrionidae). The storage pests used for the research were readied in growth chambers under control conditions (darkness, 30 ± 1 °C; relative humidity, 60 ± 5%). *T. confusum* was reared on commercially available oat flakes mixed in equal proportion with oat flour. The biotests were carried out in the same climatic chambers.

To test the feeding-deterrent activity and the lethal effect of goldenrod extract, the wheat-wafer disk bioassay was used. The wheat-wafer disk bioassay is a common method used in entomology to test the feeding-deterrent activity of various compounds on insects. This method was first described by Nawrot et al. 1986 [31] and later in 2009 [32], and it has been replicated and used consistently in research, as indicated by its adoption in studies such as Jackowski et al. [61,62]. The following concentrations of goldenrod methanol extract were used for the biotests: 0.5 mg/mL, 1.0 mg/mL, 2.5 mg/mL, 4.0 mg/mL, 5.5 mg/mL, 6.0 mg/mL, and 7.0 mg/mL. These extract doses were selected based on toxicity tests of the other *Solidago species*—namely, *S. graminifolia* ethanol extract on maize pest *Spodoptera frugiperda* [22]. In this study, the authors showed a half-maximal lethal concentration (LC_50_) of 0.496 mg/mL. Therefore, the test doses of *Solidago gigantea* methanol extract were determined from a concentration of 0.5 mg/mL.

To prepare individual concentrations, 99% ethanol (as a solvent) and one drop of tween 80 (as an emulsifier) were used. As a control, 99% ethanol and one drop of tween 80 was used. Experiments were carried out in series (extract with a relevant concentration versus a control), so each time, control samples were prepared. Wheat-wafer discs with a diameter of 1 cm were immersed in a relevant solution of the tested extract or the solvent (99% ethanol with one drop of tween 80) using pincers and placed in large glass Petri dishes (diameter 210 mm). After 30 min of drying, the discs were weighed (accuracy up to 0.01 mg) and placed in polystyrene Petri dishes with a diameter of 90 mm. When the weighing of the disks had been completed in all treatments, the test insects, whether male or female, were placed in Petri dishes, which were then covered with lids and placed in the growth chamber (conditions described above). The tests were carried out in five replicates. Insects used for research were selected from breeding farms in the pupal stage, allowing for sex division. The experiment was carried out in a climatic chamber under constant conditions (darkness, 30 ± 1 °C; relative humidity, 60 ± 5%). In a single replicate, five adults—2–5-day-old male or female of *T. confusum*—were used. After 120 h, the pests were removed from the Petri dishes. Insect mortality was checked, and the remaining wheat wafers were weighed again.

### 4.5. Assessment of the Effects of S. gigantea Aqueous Extract on Plant Growth

To assess the effect of the aqueous extract of giant goldenrod (*Solidago gigantea*) on seed germination and plant growth, tests were conducted using the following model plants: winter wheat (*Triticum aestivum* L.), field cucumber (*Cucumis sativus* L.), and cress (*Lepidium sativum* L.). The seeds were surface-disinfected with 5% NaOCl for 15 min, then rinsed three times with sterile distilled water. The aqueous extract was prepared by evaporating the methanolic extract to dryness using a rotary vacuum evaporator. The dried methanolic residue was weighed and subsequently dissolved in water containing 0.05% Tween 20 at 40 °C, with gentle agitation to ensure complete solubility.

#### 4.5.1. Seed Germination Assay

The disinfected seeds were soaked for 15 min in an aqueous extract of goldenrod at concentrations of 2.5, 5, and 10 mg/mL. After drying, the seeds were placed on 0.5% water agar (10 tubes for each dose). Seeds soaked in distilled water served as a control. After 7 days of incubation at room temperature (daylight), the length of the above-ground parts of the seedlings (in cm) was measured. Subsequently, plant biomass was determined by drying the plant material at 50 °C for 24 h and additionally at 105 °C for 1 h. The plants were weighed (10 replicates), and the results are presented as a percentage of the control. Seedling length was measured after 7 days of growth. The results are presented in Figure 8 and Figure 9.

#### 4.5.2. Plant Growth Assay at the Leafy Stage

The effect of the aqueous extract of *Solidago gigantea* on plants during the early developmental stages was assessed in vivo. The experiment was conducted in a phytotron under controlled environmental conditions: photoperiod of 16/10 h (day/night) and temperature of 26/18 °C. The seeds were planted in the soil, and after emergence, in the early vegetative phase, the plants were sprayed with an aqueous extract of goldenrod at concentrations of 10, 5, and 2.5 mg/mL in an amount of 2 mL per pot (3 replicates). Brown soil mixed with sand in a 2:1 ratio (pH 6–6.5) was used as the substrate. Spraying was performed three times—on days 10, 15, and 20 of vegetation. Plants sprayed with distilled water served as controls. The allelopathic effect was assessed using the phytotoxicity index (Table 8) after each spraying [28,29]. After 25 days of vegetation, the biomass of the above-ground parts was determined as described above. The results are presented in Table 7.

### 4.6. Statistical Analysis

All results in the tables are presented as mean ± ED (standard error) relative to the control. The applied statistical methods were two-way ANOVA and Tukey’s post hoc test. The point of significance was established as * *p* < 0.05. Statistical analyses were performed with Statistica v.13 software.

In the wheat-wafer disk bioassay (determining insecticidal potential of giant goldenrod methanol extract), mortality and loss of wheat-wafer mass (feeding inhibition) were used for statistical analyses. Mortality was calculated according to the following formula:

Mortality = (number of individuals dead after 120 h/number of individuals tested) × 100%. The effect on feeding inhibition was estimated based on the loss of wheat wafer mass after 120 h of feeding.

Datasets for individual concentrations and pest sex were checked for normality based on the Shapiro–Wilk W test. It turned out that the data did not have a normal distribution, so the nonparametric method (the Mann–Whitney U test) was used for pairwise comparisons (extract with selected concentration versus control). The research was not conducted for individual extract concentrations at the same time; therefore, a separate control was prepared for each test. Significance was evaluated at *p* ≤ 0.05. The analyses were performed using STATISTICA software v. 13 [TIBCO Software Inc., Palo Alto, CA, USA].

## 5. Conclusions

Plant extracts are valuable sources of bioactive compounds and represent promising alternatives to conventional pesticides, particularly in the context of rising antimicrobial and pest resistance. Extracts of *S. gigantea*, which is rich in chlorogenic acid, rutin, and quercetin derivatives, showed strong antibacterial activity against Gram-positive bacteria and bacteriostatic effects against selected plant-pathogenic Gram-negative strains, while others remained unaffected. The extract exhibited fungistatic activity, particularly against *Fusarium* spp., without demonstrating fungicidal effects. It also showed insecticidal potential against *T. confusum*, causing substantial mortality and feeding inhibition. Allelopathic activity varied among plant species, ranging from moderate effects on cucumber and winter wheat to strong inhibition in cress. Overall, *S. gigantea* appears to be a promising source of natural compounds for plant protection and pest management. Further studies on isolated compounds are required to clarify mechanisms of action, specificity, and safety.

## Figures and Tables

**Table 1:** Phenolic acid and flavonoid contents (%) of *S. giantea* methanolic extracts.

Chemical Group	Compound	Contribution [%] *
Phenolic acids	Chlorogenic acid	58
	Ferulic acid	14
	Protocatechuic acid	13
	Caffeic acid	7
	Syringic acid	5
	Coumaric Acid	3
	Vanillic acid	Tr
Flavonoids	Rutin	55
	Quercitrin	26
	Quercetin	12
	Neohesperidin	5
	Kaempferol	2
	Taxifolin	Tr
	Polydatin	Tr
Phenolic alcohol	Coniferyl alcohol	Tr

*—within the chemical group; Tr—trace (below LOQ).

**Table 2:** Bacteriostatic (MIC_50_ and MIC_90_) and bactericidal (MBC) activity of *Solidago gigantea* extract (mg/mL) against tested plant and mammalian pathogens.

		MIC_50_	MIC_90_	MBC
		**Plant pathogens**		
1.	*Burkholderia cepacia* IOR 2151	10.27 ± 0.11 ^d^	16.60 ± 0.21 ^c^	˃10
2.	*Dickeya zeae* IOR 2243	5.18 ± 0.14 ^bc^	8.66 ± 0.05 ^ab^	5 RC
3.	*Pectobacterium carotovorum* IOR 1822	6.78 ± 0.12 ^cd^	10.46 ± 0.21 ^abc^	5 RC
4.	*Dickeya chrysanthemi* IOR 1452	6.71 ± 0.20 ^cd^	9.94 ± 0.24 ^abc^	5 RC
5.	*Pseudomonas syringae* IOR 2260	1.18 ± 0.24 ^a^	4.58 ± 0.66 ^a^	5 RC
6.	*Pseudomonas syringae* var. *lachrymans* IOR 2183	4.49 ± 0.14 ^b^	8.48 ± 0.16 ^ab^	5 RC
7.	*Rhizobium radiobacter* IOR 2188	1.02 ± 0.14 ^a^	3.91 ± 0.03 ^a^	5 RC
		**Mammalian pathogens**		
8.	*Staphylococcus aureus* PCM 566	2.27 ± 0.21 ^ab^	7.61 ± 0.26 ^ab^	10
9.	*Staphylococcus pseudintermedius* PCM 2405	3.85 ± 0.24 ^abc^	9.27 ± 0.27 ^abc^	10
10.	*Bacillus subtilis* PCM 2021	1.23 ± 0.08 ^a^	7.87 ± 0.23 ^ab^	10
11.	*Escherichia coli* PCM 2561	˃10 ^e^	˃10 ^e^	˃10

MIC_50_ and MIC_90_—Minimum Inhibitory Concentration of 50% and 90% of cells, respectively; MBC—Minimum Bactericidal Concentration; ˃ no bacteriostatic or bactericidal effect within the tested range of doses; ± standard error (SE); RC—cell growth reduction. Values in columns marked with the same letters are not significantly different (Tukey HSD, *p* < 0.05).

**Table 3:** Growth inhibition/stimulation coefficient (H/S%) of saprotrophic and polyphagous plant-pathogenic fungal and oomycote colonies on Czapek medium supplemented with *Solidago gigantea* extract.

		Dose of Extract (mg/mL)			
Lp.	Tested Strains	2.5	5	10	Y
1.	*M. mucedo* Mm1	29.0 ± 1.6 ^f^	44.1 ± 0.8 ^bc^	54.7 ± 0.7 ^a^	42.3 ^C^
2.	*T. harzianum* Th1	44.4 ± 2.7 ^abc^	46.9 ± 1.0 ^abc^	46.1 ± 0.4 ^abc^	45.8 ^BC^
3.	*B. cinerea* Bc1	−0.8 ± 2.3 ^g^	2.3 ± 0.6 ^g^	7.8 ± 04 ^e^	3.1 ^F^
4.	*Ph. cinnamoni* IOR 2080	35.1 ± 0.3 ^def^	37.5 ± 0.0 ^de^	40.5 ± 0.3 ^c^	37.7 ^CD^
5.	*Rh. solani* F93	0.4 ± 0.5 ^g^	14.6 ± 0.7 ^f^	22.8 ± 0.2 ^d^	12.6 ^E^
6.	*F. graminearum* IOR 722	41.5 ± 0.7 ^bcd^	45.2 ± 0.3 ^abc^	53.9 ± 0.0 ^ab^	46.9 ^BC^
7.	*F. graminearum* IOR 1970	41.0 ± 0.3 ^bcd^	41.9 ± 0.2 ^bcd^	51.2 ± 0.5 ^ab^	44.6 ^BC^
8.	*F. culmorum* Fc1	31.0 ± 0.3 ^ef^	33.3 ± 0.3 ^e^	40.5 ± 0.3 ^c^	34.9 ^D^
9.	*F. culmorum* IOR Fc5(79)	49.0 ± 0.2 ^ab^	53.3 ± 0.3 ^ab^	54.2 ± 0.2 ^ab^	52.2 ^A^
10.	*F. culmorum* IOR Fc6(1596)	49.8 ± 0.2 ^ab^	51.5 ± 0.5 ^ab^	55.2 ± 0.3 ^a^	52.1 ^A^
11.	*F. culmorum* IOR Fc12(8)	38.2 ± 0.2 ^cde^	42.3 ± 0.4 ^bcd^	43.6 ± 0.2 ^bc^	41.4 ^C^
12.	*F. culmorum* Fc16	52.0 ± 0.2 ^ab^	54.8 ± 0.2 ^ab^	56.0 ± 0.2 ^a^	54.4 ^A^
X		34.2 ^C^	39.0 ^B^	43.8 ^A^	

±—standard error (SE); X and Y—mean antifungal effect according to the tested species and dose. The mean X and Y values and the values in columns marked with the same lower/uppercase letters are not significantly different according to Tukey’s HSD test (*p* = 0.05).

**Table 4:** Growth inhibition/stimulation coefficient (I/S%) of saprotrophic and polyphagous plant pathogenic species on Czapek-Dox medium without *Solidago gigantea* extract after reisolation.

		Dose of Extract (mg/mL)			
Lp.	Fungal Strains	2.5	5	10	Y
1.	*M. mucedo* Mm1	0.0 ± 0.0 ^a^	0.0 ± 0.0 ^c^	0.0 ± 0.0 ^de^	0.0 ^F^
2.	*T. harzianum* Th1	0.4 ± 0.2 ^a^	−12.5 ± 0.0 ^d^	−6.7 ± 0.2 ^e^	−6.3 ^G^
3.	*B. cinerea* Bc1	−31.6 ± 0.3 ^b^	−33.1 ± 0.5 ^e^	−32.9 ± 0.3 ^f^	−31.9 ^H^
4.	*Ph. cinnamoni* IOR 2080	5.1 ± 1.0 ^a^	7.3 ± 0.3 ^bc^	18.1 ± 0.3 ^abc^	10.2 ^BCD^
5.	*Rh. solani* F93	5.9 ± 0.0 ^a^	0.0 ± 0.0 ^c^	−0.4 ± ±0.0 ^de^	1.8 ^EF^
6.	*F. graminearum* IOR 722	4.0 ± 0.9 ^a^	4.0 ± 0.9 ^bc^	11.5 ± 1.1 ^c^	6.5 ^CD^
7.	*F. graminearum* IOR 1970	5.1 ± 0.9 ^a^	6.7 ± 1.7 ^bc^	15.9 ± 0.7 ^bc^	9.2 ^CD^
8.	*F. culmorum* IOR Fc1	3.8 ± 0.5 ^a^	20.1 ± 1.7 ^a^	28.9 ± 0.8 ^a^	17.6 ^AB^
9.	*F. culmorum* IOR Fc5	7.7 ± 1.4 ^a^	13.6 ± 3.1 ^ab^	24.3 ± 0.8 ^ab^	15.2 ^AB^
10.	*F. culmorum* IOR Fc6	2.2 ± 0.5 ^a^	4.3 ± 1.0 ^bc^	10.3 ± 0.5 ^cd^	5.6 ^DE^
11.	*F. culmorum* IOR Fc12	10.1 ± 1.9 ^a^	7.7 ± 0.5 ^bc^	16.0 ± 1.1 ^bc^	11.2 ^BCD^
12.	*F. culmorum* IOR Fc16	6.1 ± 1.8 ^a^	8.9 ± 0.7 ^abc^	16.1 ± 1.0 ^c^	10.4 ^BCD^
X		8.5 ^A^	2.3 ^B^	1.6 ^B^	

(±)—standard error (SE); X and Y—mean biocidal effect according to the species and dose. The mean X and Y values and values in columns marked with the same lower/uppercase letters are not significantly different according to Tukey’s HSD test *p* = 0.05.

**Table 5:** The results of paired comparisons (Mann–Whitney U test). Influence of *Solidago gigantea* extract on *Tribolium confusum* mortality and loss of wheat-wafer mass after 120 h of *T. confusum* feeding. Significant differences marked by * (*n* = 5).

Extract Concentration (mg/mL)	Sex	Mortality		Wheat-Wafer Mass Loss	
		Z	*p*	Z	*p*
7.0	Female	2.3	0.02 *	1.46	0.14
	Male	0.35	0.72	−0.627	0.53
6.0	Female	0.24	0.81	1.67	0.09
	Male	2.31	0.02 *	−0.21	0.83
5.5	Female	02.04	0.04 *	−1.46	0.14
	Male	1.81	0.07	−0.63	0.53
4.0	Female	1.3	0.19	−0.84	0.4
	Male	2.34	0.02 *	−1.04	0.3
2.5	Female	−0.42	0.68	0	1
	Male	2.34	0.02 *	−2.09	0.04 *
1.0	Female	1.1	0.27	0.42	0.68
	Male	1.2	0.23	0	1
0.5	Female	0.65	0.52	01.04	0.3
	Male	1.73	0.08	−2.3	0.02*

**Table 6:** Effect of aqueous *Solidago* extract spray on plant growth evaluated by the phytotoxicity index.

Plant	Day of Vegetation	Control	2.5 mg/mL	5 mg/mL	10 mg/mL
Cucumber	15	0	0	1	1
	20 and 25	0	0	0	0
Wheat	15	0	0	1	1
	20 and 25	0	0	0	0
Garden cress	15	0	0	0	0
	20 and 25	0	0	0	0

**Table 7:** Effect of aqueous *Solidago* extract spray on plant growth relative to the control (100%).

	Control (100%)	2.5 mg/mL	5 mg/mL	10 mg/mL	
Plant	Biomass (Gram)	% of Control			X
Cucumber	1.7	137 ± 2.8 ^b^	112 ± 4.1 ^a^	96 ± 2.7 ^a^	115 ^C^
Winter wheat	2.1	96 ± 1.4 ^ab^	111 ± 3.6 ^b^	91 ± 0.6 ^a^	99 ^B^
Garden cress	0.29	91 ± 4.6 ^b^	75 ± 2.3 ^b^	55 ± 4.8 ^a^	74 ^A^
Y		108 ^B^	99 ^B^	81 ^A^	

±—standard error (SE); X and Y—mean effect of the extract according to plant species and applied dose. Mean values for X and Y, as well as values in rows marked with the same lowercase or uppercase letters, do not differ significantly according to Tukey’s HSD test (*p* = 0.05).

**Table 8:** Index scale for evaluating the phytotoxic effect.

Index (H/S, %)	Effect on Leaves and Growth
0	No effect; plant appears healthy
1	Slight growth inhibition; mild chlorosis on leaves
2	Moderate growth inhibition; chlorotic or necrotic spots on leaves
3	Strong growth inhibition; visible morphological changes and necrosis of leaf portions
4	Complete growth inhibition; severe leaf damage and plant death

## Data Availability

Data will be made available upon request.

## Abbreviations

The following abbreviations are used in this manuscript:

AMR	Antimicrobial Resistance
CGA	Chlorogenic Acid
GAE/g	Gallic Acid Equivalents per gram
LD_50_	Lethal Dose at 50%
MIC_50_	Minimum Inhibitory Concentration for 50% of isolates
MIC_90_	Minimum Inhibitory Concentration for 90% of isolates
MBC	Minimum Bactericidal Concentration
TFC	Total Flavonoid Content
TPC	Total Phenolic Content
